# Sus_circPAPPA2 Regulates Fat Deposition in Castrated Pigs through the miR-2366/*GK* Pathway

**DOI:** 10.3390/biom12060753

**Published:** 2022-05-26

**Authors:** Ximing Liu, Ying Bai, Ran Cui, Shuaihan He, Xingbo Zhao, Keliang Wu, Meiying Fang

**Affiliations:** 1 National Engineering Laboratory for Animal Breeding, MOA Laboratory of Animal Genetics and Breeding, Department of Animal Genetics and Breeding, College of Animal Science and Technology, China Agricultural University, Beijing 100193, China; lximing2018@163.com (X.L.); ranc@cau.edu.cn (R.C.); heshuaihan@cau.edu.cn (S.H.); zhxb@cau.edu.cn (X.Z.); liangkwu@cau.edu.cn (K.W.); 2 College of Life Sciences and Food Engineering, Hebei University of Engineering, Handan 056021, China; baiy1986@cau.edu.cn; 3 Sanya Institute of China Agricultural University, Sanya 572025, China

**Keywords:** pig, circular RNA, castration, fat deposition, testosterone deficiency

## Abstract

CircRNAs play an important role in fat deposition, and testosterone-deficient boars exhibit significantly increased fat deposition; however, the mechanism by which testosterone regulates fat deposition through circRNAs remains unclear. In this study, circRNA-seq of backfat and abdominal fat from castrated and intact full-sib Yorkshire pigs was performed. The GO and KEGG enrichment analyses revealed that the host genes of the dorsal DE circRNAs were mainly involved in fatty acid transport, while in abdominal tissues, these genes were mainly involved in adipogenesis and inflammation. The interaction among sus_circPAPPA2, ssc-miR-2366 and *GK* was verified by dual fluorescence experiments and in porcine preadipocytes. The overexpression of sus_circPAPPA2 significantly inhibited the differentiation of preadipocytes. The expression of sus_circPAPPA2 was increased after adding 100 nM of testosterone, and preadipocyte differentiation was significantly inhibited. Testosterone can affect preadipocyte differentiation by upregulating the expression of sus_circPAPPA2, sponging miR-2366 and regulating the expression of genes, such as *GK*. These results indicate that testosterone can regulate the expression of adipocyte differentiation- and lipid metabolism-related genes by regulating the expression of circRNA, and ceRNA networks are different in the testosterone regulation of adipose deposition in different parts. This study provides basic data enhancing the understanding of the interaction between the hormone environment and mir-2366/GK to regulate trait performance in pigs.

## 1. Introduction

Castration is commonly used as a means of removing odors from boar pork. However, the castration of boars results in a significant increase in carcass fat content and causes metabolic symptoms (MS) such as central obesity, insulin resistance and glucose intolerance [1]. Studies have shown that adipose tissue deposition is affected by multiple factors, including hormonal and endocrine regulation, which are important components [2,3]. Castrated boars have significantly higher fat content and lower serum testosterone than intact boars [4,5,6]. Numerous clinical and animal experiments have confirmed that low testosterone levels can lead to massive fat deposition in the body [1,7] and that testosterone deficiencies in men can lead to significant abdominal obesity and related metabolic disorders [8]. In addition, testosterone plays an important role in regulating the distribution of fat in the body [9]. Since there are differences in glucose and lipid metabolism and endocrine function across different parts of adipose tissue [10,11], through the depot-specific impact on the cells of each fat compartment, testosterone could modulate body fat distribution patterns [12]. However, knowledge regarding the specific molecular mechanisms by which testosterone affects fat deposition and adipose tissue at different sites is limited.

Currently, with the development of sequencing technologies, an increasing number of novel non-coding RNAs are being discovered, and their regulatory roles in genes involved in cellular functions and metabolism are also known [13,14]. Among them, circular RNAs (circRNAs) can form a covalently closed continuous loop that is less susceptible to degradation by exonuclease and is more stable than linear RNAs. Most circRNAs are derived from exons, while a small portion are formed by the direct circularization of introns. Some circRNA molecules contain miRNA response elements (MREs), which can act as competing endogenous RNAs (ceRNAs) that bind miRNAs and act as miRNA sponges in cells. Then, the inhibition of miRNA on target genes is relieved, and the expression level of target genes is upregulated [15]. Some studies have shown that circRNAs can affect adipose tissue transformation and energy metabolism, rendering them a promising target for anti-obesity therapy [16,17,18]. The differences in adipogenic differentiation and lipid metabolism among different pig breeds may be due to different circRNAs [17]. During the different differentiation stages of Chinese Erhualian pigs, the expression of circRNAs was changed in subcutaneous fat tissues [18]. In chickens, circRNA may affect adipogenesis by regulating miRNA-, PPAR- and fatty acid metabolism-related pathways, such as circLCLAT1, circFNDC3AL, circCLEC19A and circARMH1 [19]. In ducks, circ-PLXAN1 can affect the differentiation of duck adipocytes, which regulate the corresponding genes and participate in fat deposition by binding miR-214 [20]. In buffalo, circRNAs 19:45387150|45389986 and 21:6969877|69753491 might be potential regulators of fat deposition [21]. Interestingly, some circRNAs have spatiotemporally specific expression patterns, suggesting that circRNAs can be involved in the regulation of special time periods during cell or tissue development [22]. Accumulating evidence suggests that circRNAs play important roles in adipocyte differentiation [23,24].

Studies have shown that hormones can affect a variety of pathological processes by regulating the expression of corresponding circRNAs [25]. The important regulatory role of circRNAs in pig fat deposition after castration has not been reported; thus, circRNA sequencing was used to identify and compare circRNA expression in the adipose tissue of Yorkshire pigs derived from castrated and intact groups of full-sibling pigs. Our purpose is to analyze the interaction mechanism between the inner hormone environment and the key genes that regulate pig fat deposition traits. The specific mechanism by which circRNAs regulate testosterone fat deposition and their effect on fat deposition in different parts were analyzed by expression profiling and testosterone addition experiments. To control excess fat deposition in pig production and improve pig production efficiency, circRNA molecular markers were provided, which can simultaneously provide a medical model for obesity caused by low testosterone in humans.

## 2. Materials and Methods

### 2.1. Tissue Samples

In this experiment, ten full-sibling male Yorkshire piglets were selected and divided into five pairs according to the principle of the pairing design. The initial conditions of the test individuals were kept as consistent as possible, and each pair of male piglets was composed of two full-sib individuals from the same source in the same litter with similar body weights. At one week of age, one piglet was randomly selected from each pair for surgical castration, and the other non-castrated individual was subjected to a sham treatment (i.e., an incision of the same size as that in the castration operation was made in the abdomen while avoiding damage to the gonads to produce the same stress effect) as the control. All pigs were bred under the same conditions, with free access to water and feed. At 180 days of age, the phenotypes of all pigs were recorded, and slaughter sampling was performed. The dorsal adipose tissue and abdominal adipose tissue of the castrated and intact pigs (*n* = 3) were selected for circRNA-seq. Dorsal adipose tissue is the backfat tissue from ribs 6–7, and abdominal adipose tissue is the suet tissue in the abdominal cavity of pigs. All tissue samples were frozen in liquid nitrogen for further use. There were significant differences in the backfat thickness and abdominal fat weight between the castrated and intact males (*p* < 0.05). More details of the phenotype information are described in Supplementary File S1: Table S1. The Yorkshire pigs were raised by Beijing Zhongyu Pig Co., Ltd., Beijing, China.

### 2.2. RNA Isolation

The total RNA was isolated using TRIzol^®^ Reagent (Invitrogen, San Diego, CA, USA) according to the manufacturer’s instructions. The RNA quality was assessed using 1% agarose gels. The RNA purity was determined using a K5500 Spectrophotometer (Kaiao, Beijing, China). The RNA integrity and concentration were assessed using an RNA Nano 6000 Assay Kit (Agilent Technologies, Foster, CA, USA) and a Bioanalyzer 2100 system (Agilent Technologies, Foster, CA, USA).

### 2.3. CircRNA Sequencing Library Construction

The total RNA was isolated by the TRIzol Reagent (Invitrogen, San Diego, CA, USA). The RNA quality, purity, integrity and concentration were assessed using 1% agarose gels, a K5500 spectrophotometer (Kaiao, Beijing, China) and the RNA Nano 6000 Assay Kit of the Bioanalyzer 2100 system (Agilent Technologies, Foster, CA, USA). The rRNA was removed by Ribo-Zero (Epicenter, Madison, Wisconsin). After the RNase R enzymatic digestion of the linear RNA, a fragmentation buffer was added, and the fragmentation was performed to 140–160 nt. Then, the reaction products were purified and recovered using magnetic beads. The end repair reaction system was set up to allow the reaction to warm appropriately in a thermomixer for the indicated time, and the sticky ends of the cDNA duplexes obtained from reverse transcription were repaired with the use of an enzyme. The PCR products were recovered by magnetic bead purification. The recovered product was dissolved in an EB solution. After the cluster generation, the libraries were sequenced on an Illumina HiSeq^TM^ 4000 platform (Illumina, San Diego, CA, USA).

### 2.4. CircRNA Identification and Differential Expression Analysis

(1) Reads containing adapters (adapter contamination), (2) reads with an unknown base N content greater than 5% and (3) low-quality reads (bases with a quality value of less than 10 account for more than 20% of the total bases in the read as low-quality reads) were removed. The clean reads were aligned to the ribosomal database using the short read alignment tool SOAP2 (http://soap.genomics.org.cn, accessed on 1 May 2019) [26], allowing up to five mismatches. Reads with ribosomes on the alignment were removed, and the retained data were used for the circRNA prediction and quantification. The clean reads were aligned to the reference genome sequence (Sus scrofa 11.1) using BWA (Version: 0.7.12) [27]. CircRNA looping sites were identified based on back-spliced junctions (bsjs) reads using the de novo circRNA prediction software CIRI (Version: v2.0.2) [28]. The circRNA exon location information and alternative splicing events were identified using CIRI-AS software (Version: v1.2) [29]. The CircRNA expression was based on backsite-junction reads, and the counts were normalized using SRPBM (spliced reads per billion mapped reads) [30]. The significantly differentially expressed circRNAs were defined as those with a fold change ≥ 2 and *p* ≤ 0.05.

### 2.5. Functional Enrichment Analysis

The host genes and predicted target genes of the differentially expressed circRNAs were analyzed by GO annotation (http://kobas.cbi.pku.edu.cn/. Accessed on 1 January 2021) and KEGG pathway (http://kobas.cbi.pku.edu.cn/, accessed on 1 January 2021) analyses. IntaRNA (Version: intaRNA: 2.0.3), miRanda (Version: miRanda-3.3a) and PITA (Version: ViennaRNA-1.6) software were used to predict the potential microRNA targets of the circRNAs. A circRNA–miRNA network was constructed according to the prediction of the miRNA binding sites by TargetScan (Version: V6.0) and miRanda software (Version: miRanda-3.3a). Cytoscape (Version: 3.6.1) software was used to construct the circRNA–miRNA networks.

### 2.6. Validation of circRNAs by RT–qPCR

The expression levels of eight circRNAs randomly selected from the circRNA sequencing results were verified by RT–qPCR. A primer design sequence comprising a reverse cleavage site was constructed. The details of the divergent primers are shown in Supplementary File S1 Table S15 RT–qPCR was performed using SYBR Green Universal Mastermix (Tiangen, Beijing, China) on a CFX96^TM^ Real-Time system (Bio–Rad, Hercules, CA). The PCR program was 95 °C for 15 min, 40 cycles of 95 °C for 30 s, 60 °C for 30 s and 72 °C for 30 s, followed by a melting curve analysis (65–95 °C) in the last cycle to evaluate the amplification specificity. β-actin was used as an endogenous control. The expression of the circRNAs was calculated via the 2^−ΔΔCt^ method.

### 2.7. Vector Construction

The vectors required for the double fluorescence experiments and preadipocyte differentiation experiments were constructed.

According to the sequence of the targeted binding sites, psiCHECK-2 vectors (Promega, Madison, WI, USA) containing the targeted binding sites (wild type) and mutation sites (mutant type) were synthesized by Qingke (Beijing, China). For example, sus_circPAPPA2 wild-type (p-sus_circPAPPA2) and mutant (p-mut-sus_circPAPPA2) as well as *GK* wild-type (p-*GK*) and mutant (p-mut-*GK*) reporter vectors were constructed. The miRNA mimic NC was synthesized by Qingke (Beijing, China). The sequence of the mir-2366 mimic was as follows: 5′UGGGUCACAGAAGAGGGUCUGG3′.

To stabilize the overexpression of sus_circPAPPA2 in the porcine preadipocytes, according to the data obtained by our sequencing, the Plc5-cirR vector (JiSai, Guangzhou, China) overexpression vector containing the full-length fragment of sus_circPAPPA2 was synthesized by 100 nmol vectors and transfected into cells using Lipofectamine 2000 (Invitrogen, Waltham, MA, USA).

### 2.8. Dual Luciferase Validation of miRNA Binding Sites in Target Genes

According to bioinformatics software (miRanda and TargetScan), we predicted the binding sites among sus_circPAPPA2, mir-2366 and *GK*. Psicheck-2 vectors (Promega, Madison, Wisconsin, USA) containing sus_circPAPPA2 wild-type (p-sus_circPAPPA2) and mutant (p-mut-sus_circPAPPA2) reporter fragments were constructed. The *GK* wild-type (p-*GK*) and mutant (p-mut-*GK*) reporter vectors were similarly constructed. In 24-well cell culture plates, 200 nmol of reporter plasmids (p-sus_circPAPPA2, p-mut-sus_circPAPPA2, p-*GK* and p-mut-*GK*) were transfected into 293T cells along with 80 nM of mir-2366 or negative control. After 48 h, luciferase activity was detected using a Dual-Luciferase Reporter System Kit (Promega, Madison, WI, USA).

### 2.9. 293T Cell Culture and Transfection

HEK293T, a human cell line of renal epithelial cells, was used to validate the miRNA targets. Cells were seeded into 24-well plates. The cotransfection with 200 ng of target mRNA-WT or target mRNA-MUT and 10 μL of miRNA mimic or mimic-NC was performed using Lipofectamine 2000 (Invitrogen, USA). Subsequently, the luciferase activity was measured using a Dual-Luciferase Reporter Assay System (Promega, Madison, WI, USA) at 48 h post-transfection. The assays were performed in triplicate.

The 293T cells were purchased from the Cell Bank of the Chinese Academy of Sciences and cultured in RPMI-1640 (Gibco, Grand Island, NY, USA) supplemented with 10% fetal bovine serum (FBS; Gibco), 100 units/mL of penicillin (Gibco) and 100 mg/mL of streptomycin (Gibco) at 37 °C in an incubator containing 5% CO_2_ and 95% air.

### 2.10. Overexpression and Differentiation of Preadipocytes

Preadipocytes were obtained from the Jingdong Yin group. After growing to 7 days of age, the Yorkshire boars were slaughtered, and the primary preadipocytes were isolated from the dorsal adipose tissue. The preadipocytes were cultured in DMEM/F12 (HyClone, Logan, Utah) supplemented with 10% fetal bovine serum (FBS) (Gibco, Logan, Utah), 100 mg/mL of streptomycin (Life Technologies, Carlsbad, CA, USA) and 100 U/mL of penicillin (Invitrogen, Invitrogen).

To induce differentiation, the primary preadipocytes were inoculated in 6-well plates until cell fusion. Subsequently, the cells were cultured in a medium containing 1 μM of dexamethasone (Sigma, St. Louis, MO, USA), 0.25 mM of IBMX (Sigma, St. Louis, MO, USA) and 50 μg/mL of insulin (Sigma, St. Louis, MO, USA). The culture medium was changed every 2 days and the cells were frozen for future studies. The cells were further incubated for 48 h; then, the medium was replaced with a maintenance medium (a growth medium supplemented with 50 μg/mL of insulin) and the cells were incubated for an additional 48 h. Then, the cells were cultured in a growth medium until maturation at 6 days.

Testosterone (100 nM) was used to measure the effects on genes involved in preadipocyte differentiation. Based on previous studies conducted in our laboratory, preadipocyte differentiation could be significantly inhibited by 100 nm of testosterone. Testosterone (Sigma, St. Louis, MO, USA) (0.288 g) was dissolved in 10 mL of methanol (Jingke, Jiangsu, China) to create a 1 mg/mL stock solution. In the process of preadipocyte differentiation, 20 µL of storage solution were added to 19.98 mL of differentiation medium to generate a working solution with a concentration of 100 nM.

All preadipocyte experiments were performed in 6-well plates, and each treatment included three biological replicates. Four control experiments were designed by varying the testosterone content of the preadipocyte differentiation medium, including sus_circPAPPA2 + NC, NC + NC, sus_circPAPPA2 + 100 nM and NC + 100 nM. In parallel, induced differentiation experiments in the preadipocytes were performed, and the cells were collected on Day 6 of differentiation for the relevant gene quantification and oil red O staining.

### 2.11. Statistical Analysis

The data are expressed as the means ± standard deviation (SD). Significance was analyzed using a one-way analysis of variance (ANOVA) to test the homogeneity of variances via Levene’s test, followed by Student’s *t* test. The calculations were conducted using SAS version 9.0 (SAS, Cary, NC, USA). The differences were considered statistically significant at *p* < 0.05.

## 3. Results

### 3.1. Overview of circRNA Expression Profiles

**Figure 1:**
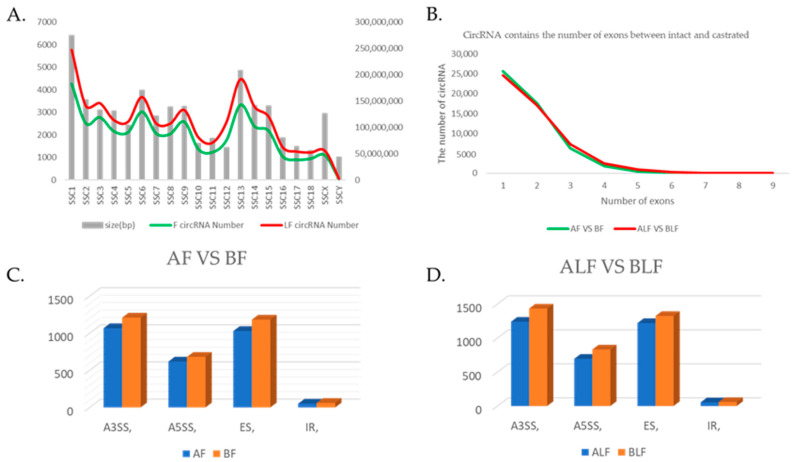
Identification of circRNAs. (**A**) Correlation between the number of circRNAs identified in adipose tissue and the chromosome size (bp). The green line represents the number of circRNAs identified in back adipose tissue, and the red line represents the number of circRNAs identified in abdominal adipose tissue. The Y-axis represents the number of circRNAs identified, and the right axis represents the size of the chromosomes. The X-axis represents the chromosome name. F represents back adipose tissue and LF represents abdominal adipose tissue. (**B**) CircRNA contains the number of exons between the intact and castrated pigs. (**C**) Analysis of the variable splicing forms of circRNAs identified in AF and BF. (**D**) Analysis of the variable splicing forms of circRNAs identified in ALF and BLF. RNA A3SS (3′ variable splicing), A5SS (5′ variable splicing), IR (intron retention) and ES (exon jumping). AF refers to intact pig backfat tissue, BF refers to castrated pig backfat tissue, ALF refers to intact pig abdominal adipose tissue and BLF refers to castrated pig abdominal adipose tissue.

After quality control and reference sequence alignment, in total, 1165 million clean reads of the backfat and abdominal fat remained (Supplementary File S1: Table S2). The average GC content was 66.10%. In total, 51,102 and 51,672 circRNAs were detected by CIRI2 in the backfat and abdominal fat, respectively (Supplementary File S1: Tables S3 and S4). CircRNAs were identified on all chromosomes in the pigs, and the relationship between the number of circRNAs produced on each chromosome and the size of the chromosomes was compared by alignment with the pig genome (Sscrofa 11.1). Among them, SSC1 (chromosome 1) was the most abundant, with 4266 circRNAs identified in the dorsal adipose tissue and 5760 circRNAs identified in the abdominal adipose tissue. The number of circRNAs identified on SSCY (chromosome Y) was lower than that on the other chromosomes (Figure 1A), with only 44 circRNAs identified in the dorsal adipose tissue and 55 circRNAs identified in the abdominal adipose tissue. It was found that the number of circRNAs generated was significantly correlated with the size of the chromosomes.

The circRNAs were classified as exonic, intronic or intergenic according to their region in the genome. According to our data, the circRNAs were mainly generated by exons (Supplementary Figure S1). A gene can produce multiple circRNAs; 85% of the circRNAs were expressed from known protein-coding genes, and most circRNAs had only one exon, followed by two exons and three exons (Figure 1B). Various circRNA variable splicing results mainly included four types of variable splicing (Figure 1C,D). Products resulting from all basic types of alternative splicing of linear RNAs can be found in circRNAs, whereas some circRNAs contain exons that are not found in linear transcripts. In addition, the amount of circRNAs produced in the castrated group was greater than that in the intact group.

### 3.2. Differential circRNA Expression Analysis between Intact and Castrated Male Pigs

**Figure 2:**
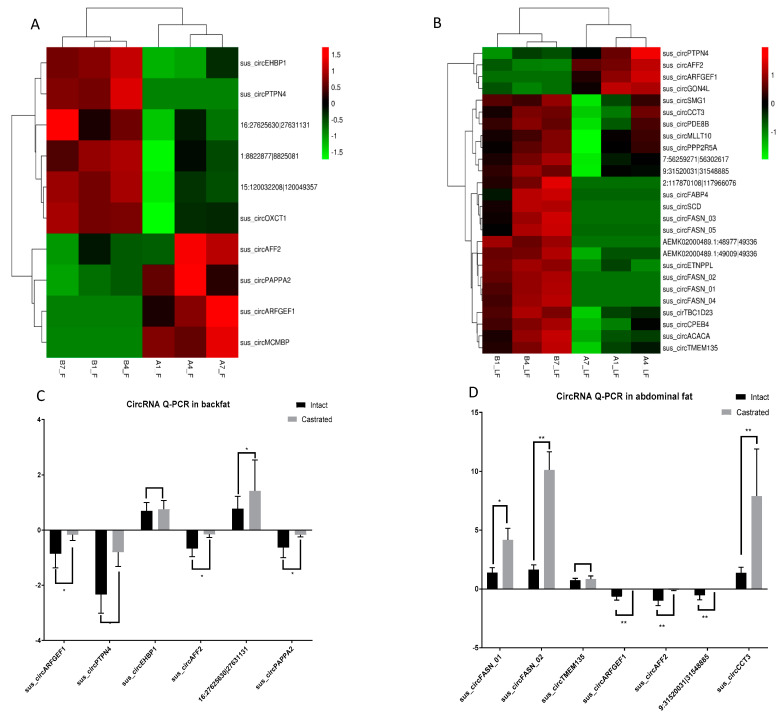
Expression profile of circRNAs. (**A**) Hierarchical cluster analysis diagram of differential circRNAs between AF and BF. (**B**) Hierarchical cluster analysis diagram of differential circRNAs between ALF and BLF. Differentially expressed circRNAs in three pairs, fold change ≥ 2.00 and *p* ≤ 0.05. (**C**) Quantification of differentially expressed circRNAs between AF and BF. (**D**) Quantification of differentially expressed circRNAs between ALF and BLF. The results are shown as the means ± standard deviation of triplicate measurements. * indicates *p* < 0.05, ** indicates *p* < 0.01. AF refers to intact pig backfat tissue, BF refers to castrated pig backfat tissue, ALF refers to intact pig abdominal adipose tissue and BLF refers to castrated pig abdominal adipose tissue.

Hierarchical clustering was performed to generate an overview of all differentially expressed circRNAs based on the values of all the expressed transcripts (Figure 2A,B). In the backfat, ten circRNAs were significantly differentially expressed between the intact and castrated male pigs, including six upregulated and four downregulated circRNAs in the castrated group compared to the intact group. In the abdominal fat, in total, 26 circRNAs, including 22 upregulated and four downregulated circRNAs, were significantly differentially expressed in the castrated male pigs. Meanwhile, two circRNAs were commonly differentially expressed in these two tissues between the intact and castrated male pigs. The two circRNAs were sus_circAFF2 and sus_circARFGEF1.

Eight differentially expressed circRNAs were further verified by RT–qPCR (sus_circPTPN4, sus_ARFGEF1, sus_ARFGEF1_2, SUS_CIRCEHBP1, 16:27625630|27631131, sus_circTMEM135, sus_circFASN_02 and sus_circFASN_01). The results of the RT–qPCR validation were consistent with those of the circRNA sequencing, except for the sus_circPTPN4 in the backfat and sus_circTMEM135 in the abdominal fat (Figure 2C,D). sus_circPTPN4 was downregulated in the backfat of the castrated male pigs compared to that in the intact male pigs but exhibited the opposite trend in the circRNA sequencing data.

### 3.3. Functional Analyses of the Host Genes of Differentially Expressed circRNAs

**Figure 3:**
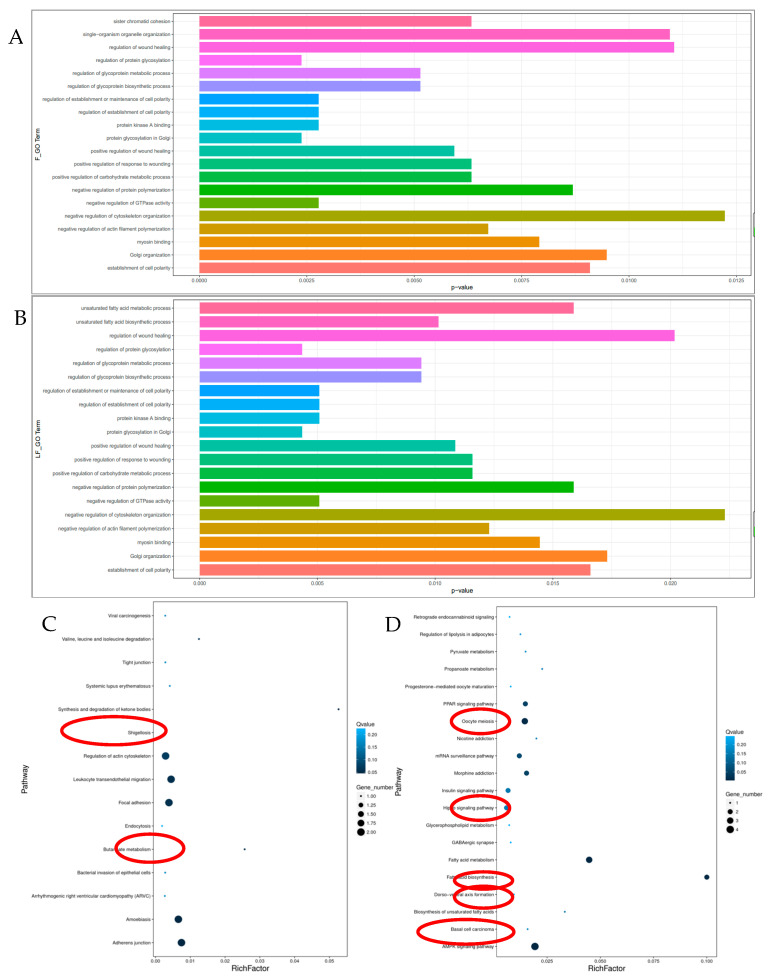
Functional analyses of the host genes of the differentially expressed circRNAs. (**A**) GO analysis of the host genes of the differential circRNAs in dorsal adipose tissue. (**B**) GO analysis of the host genes of the differential circRNAs in abdominal fat tissue. (**C**) KEGG analysis of the host genes of the differential circRNAs in dorsal adipose tissue. (**D**) KEGG analysis of the host genes of the differential circRNAs in abdominal fat tissue. Some pathways associated with fat deposition are circled in red.

To deeply explore the functions of the host genes of the differentially expressed circRNAs, GO and KEGG pathway, enrichment analyses were performed. In the backfat, the significant GO terms of the ten DEcircRNA host genes included protein glycosylation in Golgi, protein kinase A binding, regulation of establishment or maintenance of cell polarity, etc. (Figure 3A) (Supplementary File S1: Table S5) (*p* < 0.05). Regarding the KEGG analysis, the synthesis and degradation of ketone bodies and endocytosis were enriched in the backfat, which was related to fatty acid transport and metabolism (Figure 3C) (Supplementary File S1: Table S7). In the abdominal fat, the significant GO terms were mainly involved in protein glycosylation in Golgi, the positive regulation of the carbohydrate metabolic process and the fatty acid biosynthetic process (Figure 3B) (Supplementary File S1: Table S6). The KEGG pathway enrichment of the 26 DEcircRNA host genes displayed several significant pathways (*p* < 0.05), including the AMPK signaling pathway, fatty acid metabolism, fatty acid biosynthesis, insulin signaling pathway, PPAR signaling pathway and biosynthesis of unsaturated fatty acids (Figure 3D) (Supplementary File S1: Table S8). Interestingly, five DEcircRNAs in abdominal fat, sus_circFASN_01, sus_circFASN_02, sus_circFASN_03, sus_circFASN_04 and sus_circFASN_05, had the same host gene, FASN.

### 3.4. Networks and Pathway Prediction of Target miRNAs or Genes

**Figure 4:**
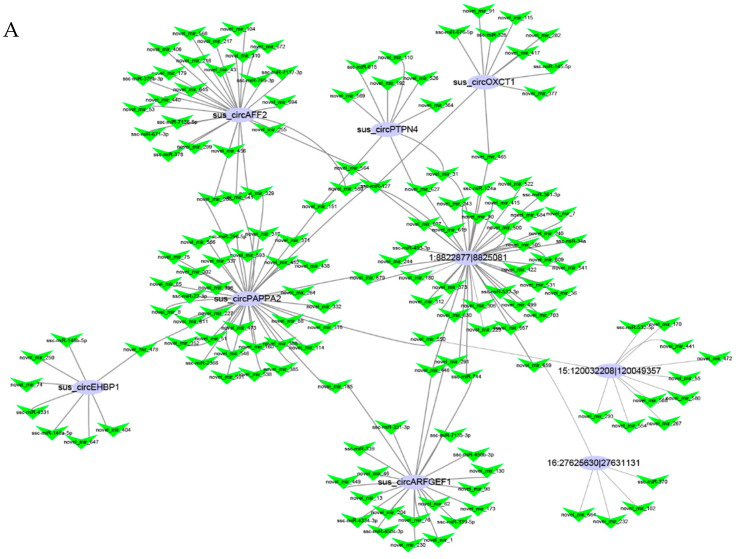

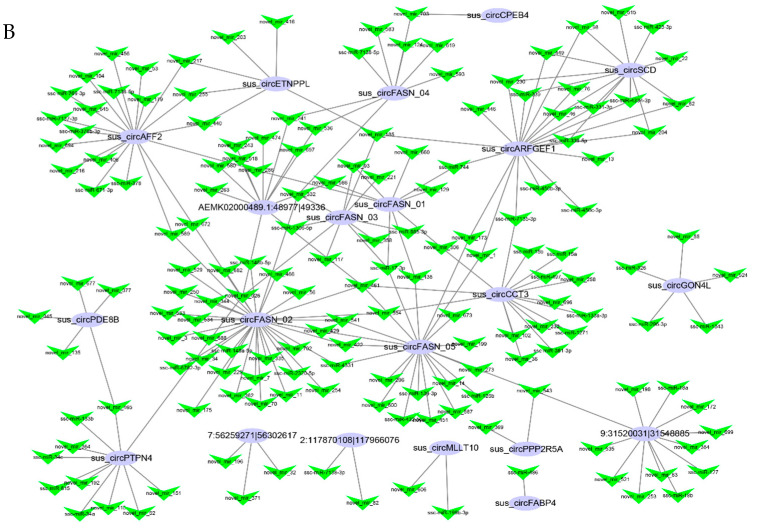
Prediction of circRNA target miRNAs. (**A**) CircRNA–miRNA network map of predicted backfat targets. (**B**) CircRNA–miRNA network map of predicted abdominal fat targets. The purple circles represent circRNAs, the green V shapes represent miRNAs and the straight line represents the interaction.

**Figure 5:**
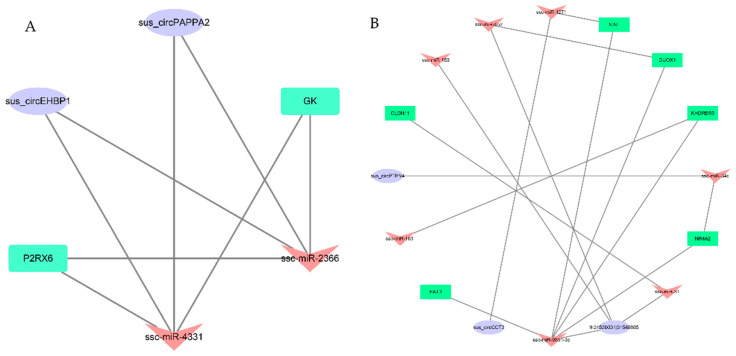
CeRNA network construction. (**A**) CircRNA–miRNA–mRNA interaction network according to the different expression data in backfat tissue between castrated pigs and intact male pigs. The network consists of two circRNAs, two miRNAs and two mRNAs. (**B**) CircRNA–miRNA–mRNA interaction network according to the different expression data in abdominal fat tissue between castrated pigs and intact male pigs. The network consists of three circRNAs, seven miRNAs and six mRNAs. The oval represents circRNAs, the V shapes represent miRNAs and the rectangle represents genes.

CircRNAs can work as miRNA sponges and regulate miRNAs. We predicted the target miRNAs of the DEcircRNAs in the backfat and abdominal fat (Supplementary File S1: Tables S9 and S10). In total, 173 and 221 targeted miRNAs were predicted according to the sequences of the 10 DEcircRNAs and 26 DEcircRNAs in the backfat and abdominal fat, respectively (Figure 4). 1:8822877|8825081 had the most nodes in the dorsal adipose tissue network, while sus_circFASN_02 had the most nodes in the abdominal fat network, with 30 overlapping miRNAs. Subsequently, circRNA–miRNA–mRNA interaction networks were constructed. Among the targeted miRNAs, three miRNAs in the backfat and 17 miRNAs in the abdominal fat that were differentially expressed between the intact and castrated male pigs according to the bioinformatics analysis and the transcriptome sequencing data were selected to construct the circRNA–miRNA–mRNA interaction networks (Figure 5). All mRNAs were also differentially expressed between the intact and castrated male pigs. There were 30 genes in the backfat and 63 genes in the abdominal fat involved in the circRNA–miRNA–mRNA interaction networks. Subsequently, a GO analysis of the putative target genes was performed to identify the potential pathways (Supplementary File S1: Tables S11 and S12). The KEGG analysis showed that 30 genes were annotated to 25 pathways (Supplementary File S1: Table S13). Among these, only the pathway involving the hematopoietic cell lineage was significantly enriched (*p* < 0.05). Sixty-three genes were annotated to 35 pathways (Supplementary File S1: Table S14), seven of which were significantly enriched (*p* < 0.05), including steroid hormone biosynthesis, the metabolism of xenobiotics by cytochrome P450, tight junction, etc.

### 3.5. The Relationship among Sus_circPAPPA2, miR-2366 and GK Was Verified by Double Fluorescence

**Figure 6:**
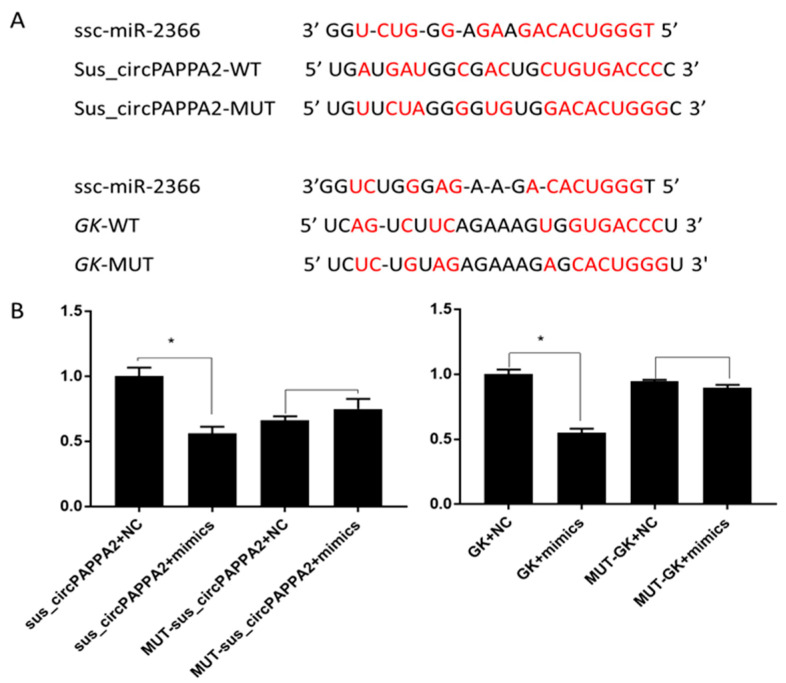
Luciferase activity following transfection with different recombinant vector plasmids. (**A**) Binding sites and mutation sites of miRNAs, circRNAs and mRNAs. Red represents the binding site of the miRNA and the corresponding fragment. (**B**) Double fluorescence was used to verify the experimental results. WT represents the wild-type fragment, MUT represents the mutant fragment and mimics represent miR-2366. * indicates *p* < 0.05, *n* = 3.

Through the ceRNA network, we selected miR-2366, sus_circPAPPA2 and *GK* to verify their interaction. The results of the double luciferase reporter gene detection system showed that the *GK* and sus_circPAPPA2 mutant vector plasmids were transfected and cloned. In the experiment, the miR-2366 group was compared with the negative control group, and there was no significant difference in the activity of luciferase (*p* > 0.05). In the transfection experiment with *GK* and the sus_circPAPPA2 vector plasmid, the luciferase activity of the miR-2366 group was significantly inhibited, and there was a significant difference in luciferase activity compared with the negative control group (*p* < 0.05). The predicted binding position and dual fluorescence results are shown in Figure 6.

### 3.6. Sus_circPAPPA2 Inhibits Preadipocyte Differentiation

**Figure 7:**
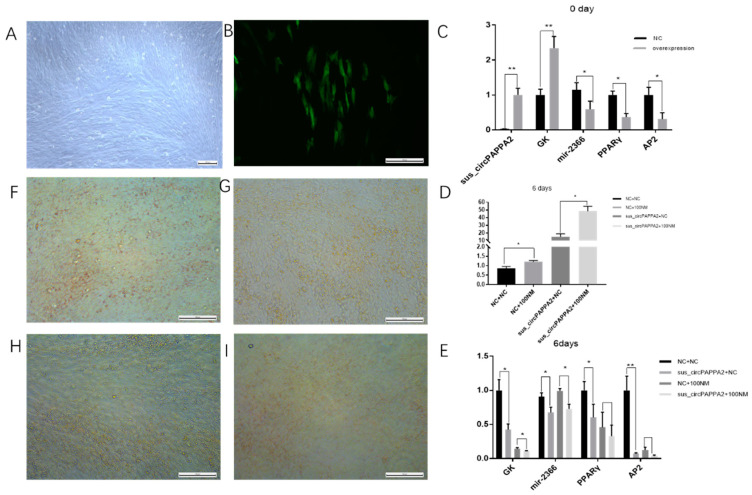
Preadipocyte differentiation experiment in the sus_circPAPPA2-overexpressing group. (**A**) Bright field image of preadipocytes overexpressing sus_circPAPPA2. (**B**) Fluorescence image of anterior adipocytes after the overexpression of sus_circPAPPA2. (**C**–**E**) qPCR quantification of related genes. (**F**) Oil red O staining was performed 6 days after differentiation in the NC group without testosterone addition. (**G**) Oil red O staining was performed 6 days after differentiation in the sus_circPAPPA2 overexpression group without testosterone addition (**H**) The NC group was differentiated with 100 nm of testosterone for 6 days after oil red O staining (**I**) The overexpressed sus_circPAPPA2 group was differentiated with 100 nm of testosterone for 6 days after oil red O staining. NC indicates the control group, sus_circPAPPA2 indicates the circRNA overexpression group, Nc + 100 nM indicates the NC group treated with 100 nm of testosterone and sus_circPAPPA2 + NC indicates the circRNA overexpression group treated with 100 nm of testosterone. * indicates *p* < 0.05, ** indicates *p* < 0.01. All pictures are magnified 100 times, *n* = 3.

To investigate the potential role of sus_circPAPPA2 in porcine preadipocyte differentiation, an overexpressing preadipocyte cell line was constructed (Figure 7A,B). Furthermore, the expression levels of *GK*, mir-2366 and other genes in the sus_circPAPPA2-overexpressing preadipocytes were determined. When the preadipocytes were undifferentiated, miR-2366 expression was decreased, and *GK* expression was increased after the overexpression of sus_circPAPPA2 (Figure 7C), which is consistent with the ceRNA relationship. We found a significant reduction in the expression of adipogenic differentiation markers in the sus_circPAPPA2 overexpression group by RT–qPCR on Day 6 after the induction of differentiation, and the oil red O staining results showed that the lipid droplet content was also reduced. The treatment with the addition of 100 nM of testosterone also resulted in a significant increase in sus_circPAPPA2, which similarly inhibited adipogenesis. The expression of mir-2366 decreased with the overexpression of sus_circPAPPA2. However, we also found that the expression of *GK*, a target gene of miR-2366, decreased in the group overexpressing sus_circPAPPA2 and the group supplemented with 100 nM of testosterone during differentiation (Figure 7D–I).

## 4. Discussion

This study analyzed the expression of circRNAs in subcutaneous and abdominal adipose tissue from intact Yorkshire pigs and castrated Yorkshire pigs for the first time. The aim of our study was to identify potential circRNAs in adipose tissue in different parts that are regulated by androgens and involved in adipogenic differentiation and lipid metabolism. The loss of testosterone is strongly associated with excessive fat deposition [2,31] and the mechanism of action of circRNAs in castration-induced fat deposition in pigs is unclear. CircRNAs have been found to have extremely important functions in various biological processes [32]. In the current study, 51,102 and 51,672 circRNAs were identified in the backfat and abdominal fat of castrated and intact pigs, respectively. The number of circRNAs identified on SSC1 was the largest, and the number of circRNAs identified on SSCY was the smallest, which is consistent with the chromosome size. Furthermore, according to the comparison of circRNAs with the reference genome, 85% of circRNAs are expressed from known protein-coding genes. This result suggests that the amount of circRNA production may be related to the size of the chromosome, and the reason for the inconsistent trend of the number of circRNAs produced by some chromosomes, such as SSCX, may be related to the number of genes, total gene length and gene activity contained in the chromosome [33,34]. In addition, the number of circRNAs in the adipose tissue from the castrated group was higher than that in the intact group, suggesting that circRNAs may play an important role in castration-induced fat deposition. The splicing form of circRNAs shows that the circRNAs from adipose tissues mainly depend on the splicing and cyclization of the end of the cord. On the mRNA precursor, a small ribosomal protein was assembled continuously to catalyze the connection between the 5′ donor site downstream of the exon and the 3′ receptor site upstream, and then loop RNA was formed by splicing the tail of the cord. Some introns on both sides of the exons of circRNAs contain reverse complementary sequences, which form RNA double-stranded bodies side by side at the splicing sites and form two different circRNAs with introns and without introns by variable splicing. Alternatively, the introns inside and on both sides of the exon can compete for RNA pairing and finally form different types of circRNA through variable splicing. This result is consistent with previous studies [35,36,37]. The exons involved in back splicing in many circRNAs tend to have long intronic flanks, and these circRNAs are generally derived from genes with highly active promoters. In addition, epigenetic alterations of histones and gene body regions that affect alternative splicing may have direct effects on circRNAs and biogenesis. Lariat formation during exon skipping (the alternative exons are spliced out of the mRNA and eventually included within the excised lariat) can lead to the formation of circRNAs when the lariat is backspliced. Finally, intronic lariats that are not hydrolyzed by lariat debranching enzymes form circRNAs. Although back splicing is viewed as one type of alternative splicing, the molecular mechanism underlying linear alternative splicing is distinct, and our data suggest that testosterones affect circRNA biogenesis in adipose tissue depending on the canonical splicing machinery.

Overweightness or obesity is a core factor of MS, especially visceral obesity, which is closely related to MS [38]. The change in visceral fat is usually the result of the change in serum testosterone, which has a positive effect on visceral fat and MS components [39]. Ten circRNAs in backfat and 26 circRNAs in abdominal fat were significantly differentially expressed between the intact and castrated male pigs. Among them, sus_circAFF2 and sus_circARFGEF1 were commonly differentially expressed. Eight differentially expressed circRNAs were further verified by RT–qPCR. The RT–qPCR validation was consistent with the results obtained from the circRNA sequencing data, except for sus_circPTPN4 in the backfat and sus_circTMEM135 in the abdominal fat. The reasons for the inconsistencies may be due to differences in the selection of individuals for the sample. More differentially expressed circRNAs were identified in abdominal fat than in backfat after castration, demonstrating that androgens have a greater impact on abdominal adipose tissue in terms of fat deposition and metabolic disorders [40].

Then, the GO enrichment and KEGG pathways of the host genes of the DEcircRNAs were analyzed. In the backfat, the synthesis and degradation of ketone bodies and endocytosis were identified, which are related to fat generation and metabolism. The gene associated with the synthesis and degradation of the ketone bodies pathway is *OXCT1* (3-oxoacid CoA-transferase 1), which is the host gene of sus_circOXCT1. *OXCT1* is a key enzyme in ketone metabolism and is highly expressed in adipose tissue [41]. The overexpression of the *OXCT1* gene in sheep adipocytes can inhibit fat formation [41]. Studies have shown that *OXCT1* activity increases in the muscles of obese mice [42]. The gene associated with the endocytosis pathway is *ARFGEF1* (ADP ribosylation factor guanine nucleotide exchange Factor 1), which is the host gene of sus_circARFGEF1. According to the GO enrichment analysis, *ARFGEF1* is involved in protein glycosylation in the Golgi and TG networks, which control lipid droplet and chylomicron formation [43]. Moreover, sus_circARFGEF1 was differentially expressed in abdominal fat and might play a key role in the regulation of abnormal lipid droplet dynamics after castration.

In abdominal fat tissues, the host genes mainly involve the AMPK signaling pathway and fat synthesis-related pathways, such as fatty acid metabolism, the biosynthesis of unsaturated fatty acids, the PPAR signaling pathway, and the insulin signaling pathway. One of them was *FASN* (fatty acid synthase), which was the host gene of sus_circFASN_02, sus_circFASN_03, sus_circFASN_04, sus_circFASN_05 and sus_circFASN_01. FASN is a key enzyme in the process of fatty acid synthesis. The amount of FASN and activity in animals have an effect on the synthesis of fatty acids and the deposition of body fat, which is of great significance for the formation of meat quality [44,45]. According to the results of the GO and KEGG analyses, the metabolism of fat deposition in backfat and abdominal fat might be different. Compared to the backfat, the abdominal fat showed a more obvious response to fat deposition caused by androgen deficiency, and the screened difference in the host genes of circRNAs was more related to fat deposition. This result is consistent with Kershaw and Jensen’s finding that visceral fat has higher levels of adrenocorticosteroids and androgen receptors and higher catabolic activity, insulin resistance and glucose uptake than subcutaneous fat and is more sensitive to adrenocorticosteroids [10,11].

Moreover, circRNA–miRNA–mRNA interaction networks were constructed. The related miRNAs and mRNAs were differentially expressed between the intact and castrated male pigs based on the results of the miRNA and RNA sequencing data (data not shown), which was verified by RT–qPCR. Among the interaction networks, in the backfat, sus_circPAPPA2 and sus_circEHBP1 formed a ceRNA relationship with ssc-miR-236, ssc-miR-4331 and the corresponding *GK* (glycerol kinase) and *P2RX6* (purinergic receptor P2X6) genes. The GK protein is a key enzyme in the regulation of glycerol uptake and metabolism. [46]. GK activity is related to blood glucose levels, insulin resistance and triglycerides [47]. Some studies proved that mir-4331 could affect energy metabolism by inhibiting RB1 expression and activating the p38 MAPK pathway, causing mitochondrial damage [48].

In the circRNA–miRNA–mRNA interaction networks of abdominal fat, the corresponding target genes are mainly involved in steroid hormone biosynthesis, the metabolism of xenobiotics by cytochrome P450 and retinol metabolism. Cytochrome P450 plays an important role in the metabolism of androgens, which are mainly involved in the metabolism of endogenous hormones, fatty acids or cholesterol [49]. Retinol-related proteins are important for androgen’s influence on visceral fat mass in mice [50]. Among them, C-9-885 and sus_circPTPN4 form a ceRNA relationship with ssc-miR-9851-3p, ssc-miR-34c and the corresponding *NR4A2* (nuclear receptor subfamily four Group A member two) genes. Nurr1/NR4A2 is an orphan nuclear receptor, and some studies indicate that unsaturated fatty acids interact with Nurr1 LBD [51]. *NR4A2* is important for regulating cell growth, metabolism, inflammation, etc. [52].

The dual fluorescence experiments proved that sus_circPAPPA2 can act as a “sponge” to adsorb ssc-miR-2366 and that mir-2366 regulates the expression of *GK.* We constructed porcine primary preadipocytes overexpressing sus_circPAPPA2 and induced them to differentiate into adipocytes. Meanwhile, we added 100 nM of testosterone as a treatment during differentiation. Through oil red O staining and marker factor detection, it was found that the differentiation of preadipocytes was significantly inhibited after the overexpression of sus_circPAPPA2. Testosterone can significantly increase the expression of sus_circPAPPA2 and inhibit the differentiation of preadipocytes. The RT–qPCR results showed altered ssc-miR-2366 and *GK* expression in adipocytes, indicating that the ceRNA network screened indeed plays an important role in the testosterone regulation of fat deposition. These results suggest that our ceRNA network may mainly play a role in the early stage of preadipocyte differentiation, while other components may be involved in the regulatory process in the late stage of differentiation. Finally, the double fluorescence experiments and overexpression of sus_circPAPPA2 in the preadipocytes indicate that testosterones affect fat deposition most likely through the sponge effect of circRNAs on genes involved in adipose differentiation. However, the circRNAs and corresponding pathways affected by testosterones are inconsistent in different sites, which is most likely direct evidence of the different modes of regulation that testosterones have on adipose tissues existing in different sites.

## 5. Conclusions

Our results provide relevant data concerning the interaction between testosterone and circRNAs that regulates fat deposition traits in pigs. We found that testosterone can regulate the expression of genes related to adipocyte differentiation and lipid metabolism by regulating the expression of circular RNAs in adipose tissue and can have different effects on fat deposition in different parts by regulating different ceRNA networks. We demonstrate that sus_circPAPPA2 is a novel circRNA that is expressed at low levels in castrated pigs and can be induced by testosterone. Mechanistically, sus_circPAPPA2 can function as a ceRNA to regulate *GK* expression by sponging miR-2366, thus inhibiting the differentiation of preadipocytes. These results provide a reference for studying the interaction between the testosterone-deficient environment and circRNAs in pigs to regulate fat deposition.

## Data Availability

The datasets analyzed during the current study are available in the NCBI BioProject database under accession number PRJNA801764.

## Supplementary Materials

The following supporting information can be downloaded: https://www.mdpi.com/article/10.3390/biom12060753/s1, Supplementary File S1 Table S1–S15; Supplementary File S2. Raw data for all the figures.

## Abbreviations

DE	Differential expression
FDR	false discovery rate
GO	Gene Ontology
KEGG	Kyoto Encyclopedia of Genes and Genomes
PCR	polymerase chain reaction
RT-qPCR	real-time fluorescence quantification
Sus_circPAPPA2	9:118402663|118403734
Sus_circEHBP1	3:78952890|78971884
Sus_circAFF2	X:120944619|120945464
C-9-885	9:31520031|31548885
sus_circOXCT1	16:26576176|26646919
sus_circARFGEF1	4:67582515|67589305
Sus_circPTPN4	15:31320424|31387416
sus_circFASN_05	12:928972|929774
sus_circFASN_01	12:929379|929774
sus_circFASN_02	12:929837|932581
sus_circFASN_03	12:932535|933265
sus_circFASN_04	12:936096|936848
sus_circTMEM135	9:20809498|20812474
